# Occipital transcranial direct current stimulation in episodic migraine patients: effect on cerebral perfusion

**DOI:** 10.1038/s41598-023-39659-5

**Published:** 2023-08-25

**Authors:** Heiko Pohl, Peter S. Sandor, Marius Moisa, Christian C. Ruff, Jean Schoenen, Roger Luechinger, Ruth O’Gorman, Franz Riederer, Andreas R. Gantenbein, Lars Michels

**Affiliations:** 1 Department of Neurology, University Hospital Zurich, Zurich, Switzerland; 2 Department of Neurology and Neurorehabilitation, ZURZACH Care, Bad Zurzach, Switzerland; 3 Zurich Center for Neuroeconomics (ZNE), Department of Economics, University of Zurich, Zurich, Switzerland; 4 Headache Research Unit, Department of Neurology-Citadelle Hospital, University of Liège, Liège, Belgium; 5 Institute for Biomedical Engineering, ETH Zurich and University of Zurich, Zurich, Switzerland; 6 Center for MR-Research, University Children’s Hospital, Zurich, Switzerland; 7 Children’s Research Center, University Children’s Hospital, Zurich, Switzerland; 8 Department of Neurology, Clinic Hietzing, Vienna, Austria; 9 Karl Landsteiner Institute for Epilepsy Research and Cognitive Neurology, Vienna, Austria; 10 Department of Neuroradiology, Clinical Neuroscience Center, University Hospital Zurich, 8091 Zurich, Switzerland; 11 Clinical Neuroscience Center, University Hospital Zurich, Zurich, Switzerland; 12 Neuroscience Center Zurich, University of Zurich and Swiss Federal Institute of Technology Zurich, Zurich, Switzerland

**Keywords:** Neuroscience, Migraine

## Abstract

Cerebral blood flow differs between migraine patients and healthy controls during attack and the interictal period. This study compares the brain perfusion of episodic migraine patients and healthy controls and investigates the influence of anodal transcranial direct current stimulation (tDCS) over the occipital cortex. We included healthy adult controls and episodic migraineurs. After a 28-day baseline period and the baseline visit, migraine patients received daily active or sham anodal tDCS over the occipital lobe for 28 days. All participants underwent a MRI scan at baseline; migraineurs were also scanned shortly after the stimulation period and about five months later. At baseline, brain perfusion of migraine patients and controls differed in several areas; among the stimulated areas, perfusion was increased in the cuneus of healthy controls. At the first visit, the active tDCS group had an increased blood flow in regions processing visual stimuli and a decreased perfusion in other areas. Perfusion did not differ at the second follow-up visit. The lower perfusion level in migraineurs in the cuneus indicates a lower preactivation level. Anodal tDCS over the occipital cortex increases perfusion of several areas shortly after the stimulation period, but not 5 months later. An increase in the cortical preactivation level could mediate the transient reduction of the migraine frequency.

**Trial registration**: NCT03237754 (registered at clincicaltrials.gov; full date of first trial registration: 03/08/2017).

## Introduction

Not just pain, but also intolerance to sound and light, nausea and vomiting, tiredness, and difficulties with concentration are hallmarks of migraine attacks^1^. Although the disorder has long attracted researchers’ interest, its pathophysiology remains incompletely understood.

Given the correlation between metabolic activity and blood flow, perfusion studies allow for the monitoring of disease activity and treatment response and thus help to deepen our understanding of the disorder^2^. Several research projects yielded essential insights into migraine attack-associated blood flow changes^3–7^. However, alterations do not only occur in the ictal phase, but also over the migraine cycle.

During the interictal period^8–11^, studies documented an increased cerebral blood flow (CBF)—amongst others—in the primary somatosensory cortex. Its extent correlates positively with attack frequency and might reflect hyper-responsiveness that—as researchers believe—takes up a central place in migraine pathophysiology^12–15^.

This hyper-responsivity may be favoured by deficient habituation of cortical responses to repeated stimuli, which itself is promoted by a lower interictal pre-excitation level of sensory cortices in episodic migraine^16^. Accordingly, excitatory neurostimulation over the visual cortex—transcranial direct current stimulation (tDCS) in particular—normalised hyper-responsivity^17^ and had a beneficial prophylactic effect in episodic migraine^18–24^.

This study investigates the cortical perfusion of episodic migraineurs and healthy controls and the influence of anodal tDCS on interictal CBF in episodic migraineurs. We hypothesised that a therapeutic modification of cortical excitability induces a reduction of hyper-responsivity that manifests as a reduced regional CBF.

## Patients and methods

### Design

This prospective, multi-centre, single-blind, randomised, and sham-controlled study consisted of a 28-day baseline period, a 28-day treatment period, and a subsequent follow-up period. Patients were randomised at the end of the baseline period to receive either active or sham tDCS treatment using a block randomisation technique with block sizes of ten participants.

Migraineurs kept headache diaries throughout the study period using the German headache diary. Moreover, we acquired magnetic resonance images from each patient at the baseline visit (BL), shortly after the stimulation period (F1) and about five months after the stimulation period (F2).

The ethics committee of Canton Zurich, Switzerland, approved the research project (registered at clincicaltrials.gov, NCT03237754, full date of first trial registration: 03/08/2017), which we conducted at the University Hospital Zurich from 2016 to 2020. All methods were performed in accordance with the relevant guidelines and regulations.

We did not predefine the sample size because no pilot data were available, but we aimed for 50 patients and 50 healthy controls (HC). The available data determine the sample size. A previous article discusses the clinical results^18^.

### Patients and healthy controls

Adult HC and migraineurs (≥ 18 years) qualified for enrolment. Eligible patients suffered from episodic migraine according to the criteria published in the third edition of the International Headache Classification^25^ and had at least two monthly migraine days (MMD) during the baseline period. Exclusion criteria were other neurologic disorders (e.g., epilepsy, stroke, traumatic brain injury, neck injury, and cerebrovascular disease), severe cardiovascular disorders (e.g., severe hypertension), and severe psychiatric disorders (e.g., acute psychosis). Moreover, we only included HC, who scored less than 11 points in the Hospital Anxiety and Depression Scale (HADS)^26^ and 0 points in the Migraine Disability Assessment (MIDAS)^27^. The latter criterion excluded all controls with an active headache disorder.

### Study treatment

During the 28-day treatment period, all migraineurs had daily treatment sessions, while HC received no treatment. With the reference electrode placed at Cz and the active electrode at Oz of the 10–20 EEG electrode placement system, participants self-applied anodal tDCS over the visual cortex using a one-channel stimulator (DCSTIMULATOR PLUS, NeuroConn, Ilmenau, Germany). All participants were trained at the baseline visit to use the device and demonstrated the instructors that they were able to use it themselves. Besides, they received an instruction manual. The current densities of the focal electrode and the reference electrode were estimated at 0.029 mA/cm^2^ and 0.01 mA/cm^2^, respectively, implying that the reference electrode had no functional effect^28^.

Each tDCS treatment session had a duration of 20 min. The stimulator maintained an intensity of 1 mA for the whole 20 min in patients receiving active tDCS and for 30 s in the sham group. During the remaining 19.5 min of the stimulation in the sham tDCS group, the device intermittently performed impedance checks suggestive of continued stimulation.

Migraineurs could use acute treatment as needed; however, changing the prophylactic medication during the study period was not permitted.

### Magnetic resonance imaging (MRI)

All imaging data were acquired on a 3-Tesla MRI Philips Ingenia scanner using a 15‐channel head coil. While we scanned migraineurs at all visits, we obtained scans from HC only once.

First, we acquired whole-brain 3D T1-weighted structural images with the following scanning parameters: 170 slices, repetition time 8.4 ms, echo time 3.9 ms, flip angle 8°, voxel dimensions 1 × 1 × 1 mm, the field of view 240 mm, and scan time 4:35 min.

Second, we acquired arterial spin-labelling (ASL) perfusion sequences with a pseudo-continuous ASL (pCASL) sequence^29^, applying the following parameters. Repetition time/time of echo 4200/16 ms, flip angle 90°, the field of view 240 mm, voxel size 3 × 3 × 6 mm, 20 slices, imaging matrix 80 × 80, labelling duration 1.65 s, post-labelling delay 1.53 s, SENSE^30^ acceleration factor 2.5, and scan duration 6:26 min. We used two pulses at 1.68 s and 2.76 s to suppress background signals and increase the signal-to-noise ratio. Subsequently, we recorded equilibrium magnetisation images (M0) with a repetition time of 10 s but otherwise unchanged pCASL sequence parameters.

### ASL analysis

First, we motion-corrected and de-noised all images using the ASLtbx toolbox (https://www.cfn.upenn.edu/zewang/ASLtbx.php)^31^, SPM 12 (The Wellcome Trust Centre for Neuroimaging at University College London) and Matlab (Version 2016b, MathWorks Inc, Natick MA). Translation of more than half a voxel size (i.e., 1.5 mm) or a rotation of more than 1° led to the participant’s exclusion. Next, the application of an isotropic Gaussian filter with a full-width-at-half-maximum (FWHM) of 6 mm reduced anatomical differences and increased the signal-to-noise ratio.

Next, we calculated CBF with the following formula.

$$ {\text{CBF}} = \frac{{60 \times 100 \times \lambda \times (M_{{{\text{control}}}} - M_{label} )}}{{2\alpha T_{1blood} \times M_{0} \left( {e^{{\frac{ - w}{{T_{1blood} }}}} - e^{{\frac{ - w + \tau }{{T_{1blood} }}}} } \right)}}. $$

Here, λ denotes the blood–brain partition coefficient for water, which we assumed to be 0.9 based on the publication of Herscovitch and Raichle^32^. M_control_ and M_label_ indicate the unlabelled baseline and the arterial spin-labelled images, respectively. M_0_ refers to the equilibrium magnetisation of blood that we calculated, multiplying the equilibrium magnetisation of CSF (measured in four distinct regions) by a correction factor for T2* decay and the blood H_2_O partition coefficient^32^. Furthermore, in agreement with the literature, we assumed a labelling efficiency $\alpha$ of 0.85^29^, a longitudinal relaxation time of blood ${T}_{1blood}$ of 1664 ms^29,33^, and set a post-tagging delay $w$ of 1.53 s. $\tau$ refers to the length of the labelling pulse train, which was 1.68 s.

Then, to obtain relative CBF values, we calculated the relative CBF dividing the subject-specific CBF-values of each voxel by the average CBF across 48 Brodmann’s areas. Finally, we normalised the CBF maps to the Montreal Neurological Image (MNI) template, provided by SPM12 (Wellcome Trust, U.K.).

### Statistical CBF analysis

The statistical analysis of the MRI data aimed to investigate whether (i) baseline CBF differs between migraineurs and HC, and (ii) active tDCS treatment leads to differences in the CBF compared with sham.

To that end, first, we compared the relative CBF at baseline of migraineurs and HC using a t-test corrected for age and sex. Then, we compared, by a repeated-measured ANOVA with the factors group (2: real and sham) and time (3: baseline, F1, and F2), the active and the sham treatment group at baseline, F1 and F2 cross-sectionally and corrected for age, disease duration, aura type (migraine with aura and migraine without aura), and the number of migraine days during the baseline period.

For these analyses (i.e. ANOVAs and t-tests), we applied a voxel-wise threshold of *p* < 0.001 and a cluster threshold of *k* = 44 according to a cluster-correction algorithm (considering the imaging matrix, recorded voxel resolution, number of slices, and spatial smoothing kernel), resulting in a p < 0.05 cluster-corrected to minimize Type I error arising from multiple comparisons^34^. We applied 1000 iterations, which refer to the number of Monte Carlo simulations, to derive the described value for *k*. The cluster threshold has been validated in the past^35^. All results are shown on a masked MNI normalized brain, to minimize the presence of potential artefacts near the scalp vicinity.

### Further statistical analyses

We report continuous variables as means and standard deviations and categorical variables as frequencies. The (non-parametric) Mann–Whitney U Test analysed the influence of a dichotomous variable on a continuous variable. Except for the MRI data analysis (see above), we used IBM SPSS statistics version 25 for the calculations and set the significance level at 0.05. Missing data are indicated at each step; they were not imputed. We did not analyse the correlation between MMD and perfusion data as the variability of the migraine days was small (see results).

### Ethics approval and consent to participate

The ethics committee of Canton Zurich, Switzerland, approved the research project. All participants provided their informed consent prior to participation.

## Results

### Clinical and demographic data

Twenty-three participants with episodic migraine (22/23 females, 95.7%) of whom 14 had migraine with aura (14/23, 60.9%), and 27 HC (21/27 females, 77.8%) enrolled in the study. During the experimental period, there was no dropout. Their average age was 38 ± 13 years and 33 ± 11 years, respectively (p = 0.107). Eleven migraineurs received active tDCS (11/23, 47.8%). The average age of participants in the active tDCS and the sham tDCS group was 41 ± 15 years and 34 ± 10 years, respectively (p = 0.288). During the baseline period, the number of migraine days did not differ statistically significantly (p = 0.134) in these groups (5 ± 3 days and 6 ± 3 days, respectively). See Table 1 for further demographic data. None of the patients experienced side effects during or after the tDCS stimulation (documented file). One subject reported problems with applying the stimulation at home, and was excluded from the study (i.e., was not part of the reported study sample).

**Table 1:** Baseline data of the included sample; all patients diagnosed with migraine with aura also had attacks without aura.

	Active tDCS (N = 11)	Sham tDCS (N = 12)	p-value	Migraine patients (N = 23)	Healthy controls (N = 27)	p-value
Age, years	41 ± 15	34 ± 10	0.288	38 ± 13	33 ± 11	0.253
Females (%)	10 (90.9)	12 (100.0)	0.478	22 (95.7)	21 (77.8%)	0.107
No. of patients with migraine with aura	7 (63.6)	7 (58.3)	> 0.999	14 (60.9)	0	–
Baseline migraine days	5 ± 2	6 ± 3	0.119	6 ± 3	0	–
Baseline headache days	7 ± 3	8 ± 2	0.107	8 ± 3	0	–
Baseline acute medication days	4 ± 3	6 ± 3	0.216	5 ± 3	0	–
Baseline total MIDAS score	19 ± 20	35 ± 23	0.031	27 ± 22	0	–
Baseline HADS-A score	7 ± 4	6 ± 3	0.382	6 ± 3	2 ± 2	< 0.001
Baseline HADS-D score	4 ± 2	4 ± 4	0.852	4 ± 3	1 ± 1	< 0.001

The number of days passed since the last attack was 7 ± 7 days in the active tDCS group and 4 ± 5 days in the sham group (p = 0.475) at the baseline visit. The number of days to the next attack was 11 ± 15 days in the active tDCS group and 4 ± 4 days in the sham group (p = 0.246, 1 missing).

F1 took place, on average, 49 ± 7 and 50 ± 9 days after the baseline examination in the active tDCS group and the sham group, respectively (p = 0.695). In the 28-days preceding F1, participants in the active group had 4 ± 3 migraine days and participants in the sham group had 6 ± 2 migraine days (p = 0.169). The number of days passed since the last attack at F1 was 5 ± 6 days in the active tDCS group and 2 ± 2 days in the sham group (p = 0.866; 1 missing). The number of days to the next attack was 10 ± 25 days in the active tDCS group and 6 ± 5 days in the sham group (p = 0.227, 1 missing).

F2 took place 210 ± 64 days and 226 ± 73 days after the active and sham group baseline examination, respectively (p = 0.347). In the 28 days preceding their examination, participants had 5 ± 4 and 7 ± 3 migraine days, respectively (p = 0.180). The number of days passed since the last attack at F2 was 14 ± 12 days in the active tDCS group and 6 ± 7 days in the sham group (p = 0.077); the number of days to the next attack was not recorded.

### Perfusion

**Figure 1:**
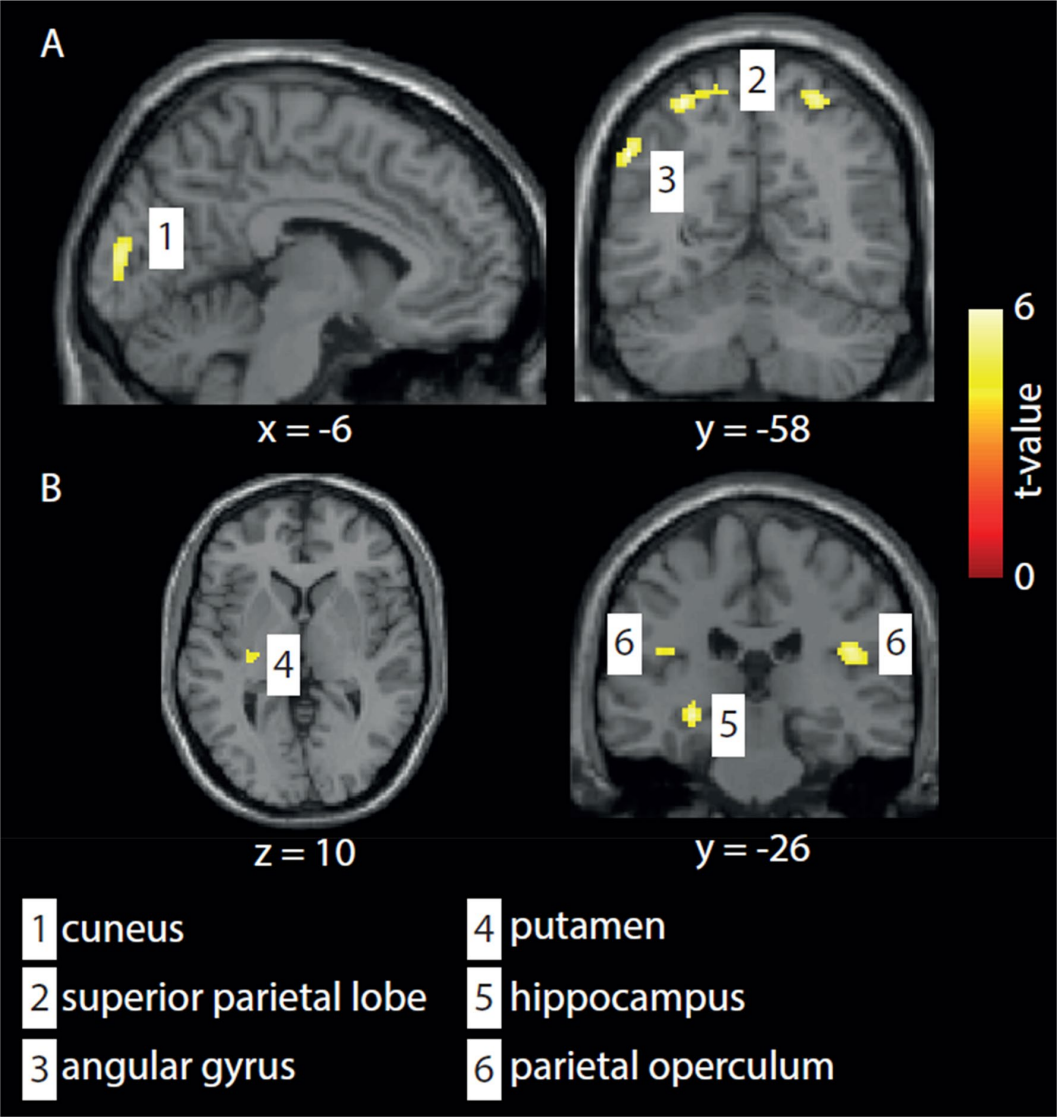
Baseline perfusion differences between all patients (sham and active tDCS) and controls. (**A**) Controls showed hyper-perfusion in the cuneus, superior parietal lobe, and angular gyrus (see Table 2). (**B**) Patients showed hyperperfusion in the parietal operculum, hippocampus, and putamen (see Table 3). Group differences are shown at p < 0.05 (cluster-corrected to minimize Type I error arising from multiple comparisons).

At baseline, HCs’ perfusion exceeded that of migraineurs in four brain areas (see Fig. 1A, Table 2). We also identified four areas in which migraineurs’ perfusion surpassed controls’, including one stimulated area (see Fig. 1B, Table 3). Baseline perfusion did not differ between migraineurs in the active tDCS group and the sham group, except one cluster in the left cerebellum exterior (MNI: − 30 − 54 − 16) for the active vs. sham group.

**Table 2:** Areas in which healthy controls’ perfusion exceeds that of migraineurs at baseline (p < 0.001, cluster threshold k = 44; analysis corrected for age, and sex) (see Fig. 1A).

Cluster size (nr. of voxels)	Peak T-value	MNI coordinates			Hemi-sphere	Region
273	5.0	26	− 64	60	Right	Superior parietal lobe
54	5.0	− 52	− 58	40	Left	Angular gyrus
119	4.9	− 8	− 92	12	Left	Cuneus
271	4.8	− 28	− 60	60	Left	Superior parietal lobe

**Table 3:** Areas in which migraineurs' baseline perfusion exceeds that of healhy controls (p < 0.001, cluster threshold k = 44; analysis corrected for age, and sex) (see Fig. 1B).

Cluster size (nr. of voxels)	Peak T-value	MNI coordinates			Hemi-sphere	Region
128	5.1	44	− 24	22	Right	Parietal operculum
47	4.6	− 28	− 26	− 8	Left	Hippocampus
195	4.2	− 24	− 16	14	Left	Putamen
66	4.4	− 40	− 30	22	Left	Parietal operculum

**Figure 2:**
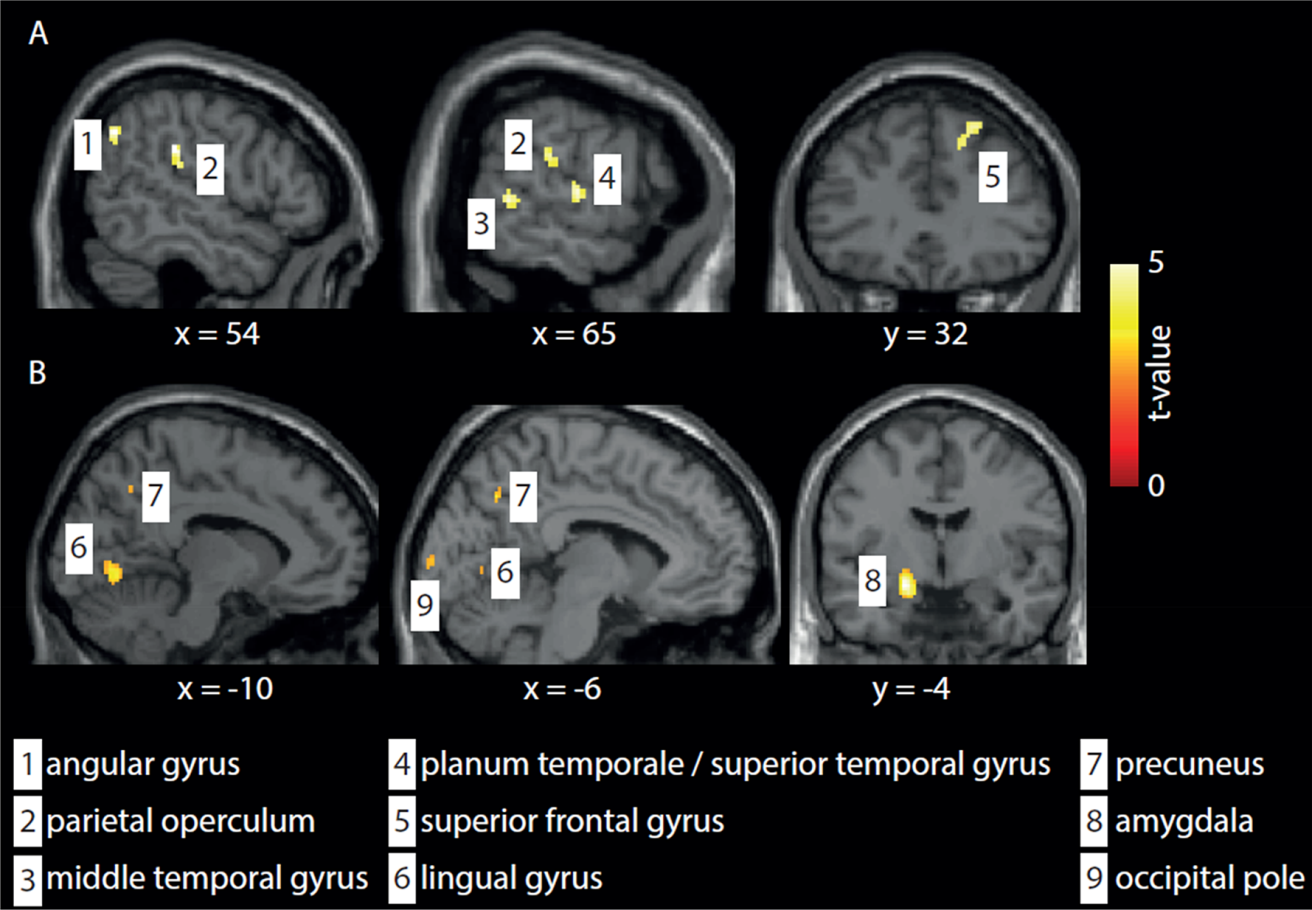
The graphic indicates post-tDCS (time point F1) perfusion in patients. (**A**) Sham > active tDCS group (see also Table 5). (**B**) Active > sham tDCS group, i.e., perfusion is increased in the lingual gyrus (see also Table 4). Results are shown with a correction for age, disease duration, aura type (migraine with aura and migraine without aura), and the number of migraine days during the baseline period. Group differences are shown at p < 0.05 (cluster-corrected to minimize Type I error arising from multiple comparisons).

Next, we compared CBF differences between the active and sham tDCS groups at different time points. There was a main effect of group (F_(1,65)_ = 11.87) and time (F_(2,65)_ = 4.94; p < 0.05 cluster-corrected) but no group × time interaction. At F1, post-hoc t-test analyses revealed that brain perfusion in patients receiving active tDCS exceeded that of patients in the sham group in seven brain areas (see Table 4, Fig. 2B). In contrast, patients receiving sham tDCS demonstrated stronger perfusion in eight brain regions, including the middle temporal gyrus (Table 5, Fig. 2A).

**Table 4:** Areas in which the perfusion of patients in the active tDCS group exceed that of those in the sham group exceeds at the first follow-up examination (F1), which was scheduled directly after the stimulation period (p < 0.001, cluster threshold k = 44; analysis corrected for age, disease duration (years), aura, and migraine days during the baseline period) (see Fig. 2B).

Cluster size (nr. of voxels)	Peak T	MNI coordinates			Hemisphere	Region
142	5.5	− 20	− 6	− 12	Left	Amygdala
341	5.2	− 34	− 36	− 24	Left	Fusiform gyrus
133	4.2	− 54	32	42	Left	Middle frontal gyrus
62	3.8	− 10	− 70	− 4	Left	Lingual gyrus
46	3.1	− 6	− 98	6	Left	Occipital pole
45	3.5	− 6	− 64	40	Left	Precuneus
98	4.2	40	− 74	26	Right	Middle occipital gyrus

**Table 5:** Areas in which the perfusion in the sham group exceeds that of patients in the active tDCS group at the first follow-up examination (F1) that was scheduled directly after the stimulation period (p < 0.05, cluster threshold k = 44; analysis corrected for age, disease duration (years), aura, and migraine days during the baseline period) (see Fig. 2A).

Cluster size (nr. of voxels)	Peak T	MNI coordinates			Hemisphere	Region
130	3.8	54	− 30	30	Right	Parietal operculum
91	3.7	54	− 60	38	Right	Angular gyrus
52	3.6	22	30	52	Right	Superior frontal gyrus
51	3.5	64	− 44	8	Right	Middle temporal gyrus
45	3.5	64	− 14	5	Right	Planum temporale/superior temporal gyrus
88	3.8	64	− 36	10	Right	Superior temporal gyrus
44	4.4	20	− 62	66	Right	Superior parietal lobe
79	4.2	44	40	20	Right	Middle frontal gyrus

At F2, there was no brain area with increased perfusion in the active group participants compared with the sham group and, conversely, no area with increased perfusion in the sham group participants compared with the active group.

## Discussion

This study analysed interictal brain perfusion of episodic migraineurs. We found that their perfusion differed in several areas that did not respect the territories of specific blood vessels. Furthermore, tDCS of the occipital lobe altered perfusion compared with sham treatment. We discuss clinical findings in an earlier article^18^.

Previous studies analysing brain perfusion in migraineurs with aura suggested hypoperfusion—or the co-occurrence of hypo- and hyperperfused areas—during the aura, followed by hyperperfusion during the headache phase that eventually normalised^4,36–38^. A recent study by Fu et al. documented both hyper- and hypoperfusion of several areas during the interictal period^39^. In patients with migraine without aura, there was hypoperfusion during the headache phase^40,41^ and hyperperfusion or no alteration during the interictal period^9,11,42^.

In our sample, we found both hypoperfusion (i.e., lower CBF in patients compared to HC) and hyperperfusion (see Tables 2 and 3). The co-occurrence of an increased and a decreased blood flow may be explained by the composition of our sample that comprised patients with and patients without aura. The lower baseline perfusion in the cuneus in patients could be the correlate of the lower preactivation level of visual cortices found interictally in migraineurs in visual evoked potential studies. These showed a lower amplitude for a low number of stimuli during the interictal period in migraineurs^19,43^ and were the rationale for applying an excitatory anodal tDCS protocol in our study.

A more detailed analysis of the areas affected by perfusion changes during the interictal period does not reveal a clear picture. Previous studies in episodic migraine patients indicated alterations in the right middle temporal gyrus^9^, or the right middle frontal orbital gyrus, and the right middle frontal gyrus^11^. Another study found no changes^42^. We localised the altered perfusion mainly in the parietal lobes.

Moreover, the functional connection between the areas with altered perfusion is unclear. Only one of the hypo-perfused areas harbours a large-scale network—the angular gyrus (see Table 2), which is a hub in the default mode network^44^. A recent study revealed increased CBF connectivity between several brain areas, none of which was hyperperfused in our sample^11^. Consequently, other yet-to-be-identified neuronal pathways must play a role.

These discrepant findings are evocative of the discussion about structural changes in migraineurs detected by voxel-based morphometry that likewise are inconsistent among studies and therefore raise the question of their validity and implications^45^. Of course, brain perfusion is not static, and the discrepant results are likely to be due to insufficient knowledge of influencing factors. For instance, perfusion is likely influenced by the time passed since the attack abated^38^. Thus, it might be more fruitful to analyse dynamic changes in cortical activity in patients and healthy controls and search for differing patterns with more advanced methods, e.g., connectome-harmonic decomposition^46,47^.

Complicating matters further, only one area listed in Tables 2 and 3—the angular gyrus—seems metabolically active during migraine attacks^48,49^. Consequently, the hyper- or hypo-perfused areas identified in this study seem unrelated to migraine pathophysiology (see Tables 2 and 3). Thus, differences between migraineurs and HC cannot be solely due to the attacks.

If cerebral perfusion correlates at least to some degree with cortical activity^15^, our findings suggest the co-occurrence of hyper- any hypo-excitability and, thus, support the idea of treating patients with tDCS. Although there was altered perfusion in many areas in our sample, the occipital gyri on which the tDCS applied in this study focused were not. Nevertheless, our clinical data indicate that tDCS resulted in a significantly reduced number of monthly migraine days^18^.

In a previous paper, we analysed the effect of tDCS on monthly migraine days in the patients enrolled in this study^18^. We found that although migraine days were significantly reduced compared with sham treatment, the effect became significant only after three months following the stimulation and quickly faded afterwards. Four months after the treatment, no statistically significant difference between the groups persisted. The time course of the clinical effects were unknown to us when scheduling F1 and F2. Since these follow-up visits took place before and after the clinical impact, we found no difference in migraine days in the 28 days preceding the MRI examinations at F1 and F2 that we analysed in this study.

At the first follow-up visit (F1), tDCS resulted in altered perfusion in frontal, temporal, and parietal cortices and the occipital pole. The hyper-perfusion in the occipital pole was expected, as anodal tDCS—which is in principle excitatory for the underlying cortex—generally induces hyper-perfusion in the stimulated area, including the cuneus^50^. The commonality between most hyperperfused regions seems to be their implication in processing and memorising visual information^51^. Thus, the stimulation effect may spread mainly through visual pathways.

The only overlap of the areas of stimulation-induced alterations of perfusion and the areas in which migraineurs’ perfusion differed from HC was the right parietal operculum. Thus, our findings do not explain how stimulation led to the clinical effect. However, as we did not scan the participants when migraine frequency differed significantly between the two groups, we cannot tell whether the alterations in perfusion had propagated to other brain areas when the effect set in.

At the second follow-up examination (F2), perfusion did not differ anymore between patients in the active and the sham tDCS groups. These findings suggest a slowly fading tDCS effect that may correlate with the reduction of the clinical effects.

### Strengths and limitations

This study has the strength of being among the first to investigate the long-term effects of self-applied tDCS on brain perfusion in episodic migraineurs and comparing these data to the migraine frequency. However, there are some limitations. First, the sample size was relatively small—we had to halt recruitment due to difficulties finding participants—and comprised migraineurs with and without aura that may have differing alterations in cortical perfusions. Second, we only included episodic migraineurs with a relatively high migraine frequency. As a result, this study will have missed changes in brain perfusion due to infrequent migraine attacks if these differ from those found in our sample. Lastly, our sample comprised mostly female migraineurs and thus may not generalise to male patients.

## Conclusions

Transcranial direct current stimulation can alter the brain’s perfusion, suggesting a correlation between blood flow and cortical responsivity. Perfusion of the occipital lobe stimulated in this study was lower in migraineurs than in healthy controls at baseline, which could be due to a lower preactivation level of visual areas. Moreover, only the right parietal operculum had been hyper-perfused in migraineurs before the treatment. Therefore, it is unknown whether direct stimulation of hyper-perfused areas would have a more pronounced clinical effect. Nevertheless, this study documents that anodal tDCS over the visual cortices in migraineurs increased perfusion. As previous statistical analysis of the clinical data showed that stimulation significantly reduced monthly migraine days in episodic migraineurs, we suggest that this could be partially mediated by an increased preactivation level, and hence increased perfusion, of the visual cortex.

## Data Availability

The anonymised data are available from the corresponding author on reasonable request. However, he will evaluate in each case whether current Swiss legislation permits data sharing.

## Abbreviations

ASL: Arterial spin labelling
BL: Baseline visit
CBF: Cerebral blood flow
F1: First follow-up visit
F2: Second follow-up visit
FWHM: Full-width-at-half-maximum
HADS: Hospital Anxiety and Depression Scale
M0: Equilibrium magnetisation images
MIDAS: Migraine Disability Assessment
MMD: Monthly migraine days
MNI: Montreal neurological image
MRI: Magnetic resonance imaging
pCASL: Pseudo-continuous arterial spin-labelling
tDCS: Transcranial direct current stimulation
